# Resveratrol protection against IL-1β-induced chondrocyte damage via the SIRT1/FOXO1 signaling pathway

**DOI:** 10.1186/s13018-022-03306-y

**Published:** 2022-09-05

**Authors:** ChuanCai Liang, Hengte Xing, ChenYu Wang, XiongFeng Xu, Yarong Hao, Bo Qiu

**Affiliations:** 1 Department of Orthopedics, Renmin Hospital of Wuhan University, Wuhan, China; 2 Department of Geriatrics, Renmin Hospital of Wuhan University, Wuhan, China

**Keywords:** Chondrocytes, Extracellular matrix, Apoptosis, Autophagy, Resveratrol, Osteoarthritis

## Abstract

**Purpose:**

Osteoarthritis (OA) is a common joint disease characterized by cartilage degeneration, synovial inflammation, osteophytes, and subchondral osteosclerosis. This study investigated the effects of resveratrol (RES) on extracellular matrix (ECM), autophagy, and apoptosis in OA pathogenesis via the SIRT1/FOXO1 pathway.

**Methods:**

The microenvironment of OA chondrocytes was stimulated in vitro by adding 10 ng/mL of IL-1β to primary Wistar rat chondrocyte. Western blotting, immunofluorescence, quantitative real-time PCR, and transmission electron microscopy (TEM) were used for analysis.

**Results:**

In the presence of IL-1β, RES increased the expression of silent information regulator (SIR) 1 protein and the phosphorylation level of forkhead transcription factor (FOXO) 1. It also promoted chondrocyte autophagy, increased the expression of SOX9 and aggrecan, inhibited chondrocyte apoptosis and matrix breakdown, and protected chondrocytes from IL-1β damage. After a SIRT1 inhibitor or FOXO1 inhibitor was added, the protective effect of RES on chondrocytes was significantly weakened. Our results suggest that RES regulates the ECM metabolism, autophagy, and apoptosis of OA chondrocytes through the SIRT1/FOXO1 pathway to ameliorate IL-1β-induced chondrocyte injury.

**Conclusion:**

RES protects chondrocytes from IL-1β-induced damage by activating SIRT1/FOXO1 signaling and holds potential in OA treatment.

## Introduction

Osteoarthritis (OA) is a chronic joint disease that can lead to chronic pain and limited joint function. Current treatment options cannot reverse OA progression and can only improve the patient’s joint pain. The degenerative cartilage change is the primary pathological change in OA [1–5]. Under normal conditions, cartilage extracellular matrix (ECM) metabolism is in a dynamic equilibrium. In OA, the activity of chondrocyte catabolic-related enzymes is increased, including matrix metalloproteinase (MMPs) and A Disintegrin and Metalloproteinase with Thrombospondin motifs (ADAMTS) [6, 7]. Lentivirus-mediated overexpression of SOX9 can downregulate the levels of some catabolic proteins, including MMP13 and ADAMTS5 [8]. Thus, maintaining or reinforcing SOX9 expression in chondrocytes can be an important therapeutic approach for regenerating cartilage tissue or delaying OA progression.

An imbalance between the autophagy and apoptosis of articular chondrocytes forms an essential part of the pathological progression of OA, where a marked increase in the level of apoptosis and a significant decrease in the level of autophagy in OA articular chondrocytes is observed [9, 10]. Autophagy is essential in maintaining normal cellular functions, mainly by regulating cellular metabolism and clearing cells with damaged organelles [11]. Therefore, regulating the balance between autophagy and apoptosis levels in chondrocytes is considered an effective approach for OA treatment.

The silent information regulator (SIR) gene family significantly affects gene silencing, genome stability, and cellular lifespan. SIRT1 promoted matrix synthesis in mouse articular chondrocytes, suggesting that it is also an intervention target for cartilage metabolism [12]. The forkhead transcription factor (FOXO) family comprises four members, FOXO1, FOXO3, FOXO4, and FOXO6. Among these, FOXO1 most heavily influences chondrocytes and has essential functions in regulating chondrocyte development, senescence, and oxidative stress [13].

Resveratrol (RES) is the strongest SIRT1 activator among polyphenolic compounds [14], and further research into the role of SIRT1 and FOXO1 in the anti-OA effect of RES is warranted. Herein, we studied the effect of RES on OA chondrocytes by simulating OA chondrocytes in vitro. The effect of RES on cartilage through the SIRT1/FOXO1 pathway provides a theoretical basis for elucidating the pathogenesis of OA and finding effective preventive and curative measures.

## Materials and methods

### Clinical cartilage specimens

Three cases of OA cartilage tissue were obtained from patients with OA who underwent knee or hip replacement surgery at the Wuhan University People’s Hospital. Two males and four females aged 61–76 years (average age 72.39 ± 3.57 years) were enrolled in the study, and normal cartilage tissue was obtained from patients who had undergone hip replacement for femoral neck fractures. All patients provided informed consent, and the research was approved by the Ethics Committee of Wuhan University.

### Handling and culturing chondrocytes

We isolated chondrocytes from the knee joints of two-week-old Wistar rats. Bilateral knee cartilage tissues were extracted and cut up using scissors in a sterile environment. The cartilage was bathed in 0.1% trypsin (Beyotime, Shanghai, China) for 60 min, followed by overnight incubation in 0.2% collagen II (Sigma, St. Louis, MO, USA) in a cell culture incubator. The digested cells were collected and resuspended in chondrocyte medium (DMEM/F-12) for culturing (BI, Kibbutz Beit Haemek, Israel). The chondrocytes in the medium were then placed in a cell culture incubator for passaging. Subsequent experiments were performed using second-generation chondrocytes. To inhibit the SIRT1/FOXO1 signaling pathway, serum-starved cartilage cells were pretreated with SIRT1 inhibitors EX-527 (10 μM/mL) or FOXO1 inhibitors AS (1 μM/mL) for 6 h and then exposed to IL-1β (10 ng/mL) in the presence or absence of resveratrol (50 μM) for 24 h.

### Transmission electron microscopy (TEM)

Cells were assigned to five groups: IL-1β, control, IL-1β + RES, IL-1β + RES + AS, and IL-1β + RES + EX-527 groups. Chondrocyte fixation was carried out with 2.5% glutaraldehyde for 3–4 h, followed by scraping with a cell scraper. Then, 2.5% glutaraldehyde was added for 2–4 h at 4 °C, followed by rinsing thrice with 0.1 M phosphate buffer. The mixture was fixed at room temperature (20 °C) for 2 h and rinsed again. Finally, dehydration, permeabilization, embedding, sectioning, and staining were performed sequentially, and electron microscopy (HITACHI, Japan/FEI, USA) was carried out.

### Immunofluorescence staining

The chondrocytes were fixed in 4% paraformaldehyde for 20 min, then washed with PBS, blotted dry, and permeabilized with phosphate Tween 20 buffer (PBST) for 20 min. Volume fraction 5% fetal bovine serum albumin was added at room temperature. Diluted primary antibody collagen (Affinity, AF0135) (1:1500) and p-FOXO1 (Affinity, AF3416) (1:1000) were added dropwise, followed by overnight incubation at 4 °C. After washing thrice, the diluted Goat Anti-Rabbit IgG antibody (Affinity, AF0135) (1:5000) was added dropwise. The samples were incubated for 1 h at 37 °C in a wet box and washed thrice with PBS. DAPI solution was added, and images were acquired using an inverted fluorescence microscope. The experiment was performed with three replications.

### Quantitative real-time PCR

The mRNA levels of aggrecan, MMP13, ADAMTS5, SOX9, SIRT1 FOXO1, LC3, ATG5, BAX, Beclin 1, caspase-3, and Bcl-2 were analyzed. TRIzol reagent (Ambion, China) was used to extract total cellular RNA. The cDNA synthesis kit (VAZYME, Nanjing, China) was used to synthesize complementary strand DNA. Target genes were amplified using the SYBR Premix ExTaq kit (VAZYME, Nanjing, China). Oligonucleotide primers used in quantitative real-time PCR are shown in Table 1.

**Table 1:** Oligonucleotide primers used in real-time PCR

Gene	Forward primer (5′-3′)	Reverse primer (5′-3′)
Rat GAPDH	5′-ACAGCAACAGGGTGGTGGAC-3′	5′-TTTGAGGGTGCAGCGAACTT-3′
Rat LC3	5′-AAAATGGGGCACGGATGAAG-3′	5′-GCAGGTCTTCAAAATGCCCA-3′
Rat MMP13	5′-CCCGAGACCTCATGTTCATCT-3′	5′-CTTCTTCTATGAGGCGGGGAT-3′
Rat Foxo1	5′-CCATGCCTCACACATCTGCC-3′	5′-TTAAAATCCAAGGTATCTCCGTCCA-3′
Rat SOX9	5′-CACAAGAAAGACCACCCCGA-3′	5′-TGCACGTCTGTTTTGGGAGT-3′
Rat Aggrecan	5′-TGACTTTCCTCCGTCTACTGTC-3′	5′-AGGTCTTCTGTGATCGGTACTC-3′
Rat ATG5	5′-GTTAGTGAGATTTGGTTTGA-3′	5′-ATTTTCTTCTGGAGGGTATT-3′
Rat Beclin 1	5′-GAGGTACCGACTTGTTCCCT-3′	5′-CCTTTCTCCACGTCCATCCT-3′
Rat Caspase-3	5′-TGGACTGCGGTATTGAGACA-3′	5′-GCGCAAAGTGACTGGATGAA-3′
Rat SIRT1	5′-TGCCATCATGAAGCCAGAGA-3′	5′-CATCGCAGTCTCCAAGAAGC-3′
Rat BAX	5′-AAGAAGCTGAGCGAGTGTCT-3′	5′-CCAGTTGAAGTTGCCGTCTG-3′
Rat Bcl-2	5′-TGACTGAGTACCTGAACCGGCATCT-3′	5′-GAGACAGCCAGGAGAAATCAAACAGA-3′
Homo GAPDH	5′-TCAAGAAGGTGGTGAAGCAGG-3′	5′-TCAAAGGTGGAGGAGTGGGT-3′
Homo SIRT1	5′-AGCAGATTAGTAGGCGGCTT-3′	5′-GACTCTGGCATGTCCCACTA-3′
Homo FOXO1	5′-TGAAACGAGCAACTATCAAAGAC-3′	5′-ATAAAAGAACCAGATGGAGGACT-3′

### Protein extraction and western blotting

The protein expression levels of SIRT1, FOXO1, ADAMTS5, SOX9, aggrecan, caspase-3, p-FOXO1, BAX, MMP13, and BCL-2 in chondrocytes were measured by the western blotting. Chondrocytes were lysed by adding cell lysis solution 24 h after cell culture, followed by protein extraction and measurement of the target protein levels using the BCA Protein Level Assay Kit (Beyoncé, China). The proteins were separated and stored at − 20 °C. Subsequently, the proteins were electrophoresed, transferred, soaked in a warm blocking solution for 1 h, and incubated overnight with the primary antibody. The membrane was washed and incubated with the secondary antibody (HRP-labeled sheep anti-rabbit, Wuhan Boster Biological Technology, LTD.) (1:50,000) for 1 h. After washing again, the proteins were detected by chemiluminescence and developed by X-ray film exposure. GAPDH was used as the endogenous protein for visualization and standardization. Densitometric analysis was conducted with ImageJ software. The process was repeated thrice under identical experimental conditions. Information on the antibodies used is listed in Table 2.

**Table 2:** Information related to antibody used in the study

Antibodies company catalog dilution ratio			
Aggrecan (250 KD)	Affinity	DF7561	1:2000
ADAMTS5 (72KD)	Affinity	DF13268	1:1000
Caspase-3 (1732KD)	Abcam	ab184787	1:2000
BAX (21KD)	Affinity	AF0120	1:2000
Bcl-2 (26KD)	Affinity	AF6139	1:1000
SIRT1 (130KD)	PROTEINTECH GROUP	60303-1-Ig	1:5000
FOXO1 (74KD)	ABclonal	A2934	1:1000
p-FOXO1 (78KD)	Affinity	AF3416	1:1000
SOX9 (56KD)	Abcam	ab185966	1:3000
MMP13 (54KD)	Abcam	Ab39012	1:3000
GAPDH (37KD)	GOODHERE	AB-P-R 001	1:1000

### Cell proliferation assay by CCK-8 kit

The influence of different RES concentrations on chondrocyte growth was assayed using the Cell Counting Kit-8 (CCK-8, MCE, China). The chondrocytes were separated into six groups and treated with 0, 25, 50, 100, 200, and 400 μM of RES, respectively. Chondrocytes were placed in 96-well plates at a concentration of 1 × 10^5^/mL and cultured in a 37 °C cell culture oven until the following day. The groups of chondrocytes were then treated with various concentrations of RES for 24 h. The CCK-8 solution was inserted into the culture wells 3 h before RES treatment and incubated for an additional 4 h. The experiment was repeated three times.

### TUNEL analysis

Chondrocyte apoptosis was examined using TUNEL analysis. Chondrocytes were divided into control, IL-1β, IL-1β + RES, IL-1β + RES + AS, and IL-1β + RES + EX-527 groups. The TUNEL staining was carried out according to the manufacturer’s instructions (Roche, IN, USA). Apoptotic cells were imaged and measured under a fluorescence microscope. The number of positive cells was quantified in three randomly selected fields of view of three sections from each sample using Image J 8.0.

### Reagents

Resveratrol (RES, no. R107315, CAS: 501-36-0, purity 99%), selisistat (EX-527, no. S1541, 99% purity), AS1842856 (AS, no. 88222, 99% purity) were purchased from Maclean Biotech (Shanghai, China).

### Statistical analysis

All experiments were repeated thrice. Data are expressed as the mean ± SEM. Graphs were generated using GraphPad Prism 8.0 (GraphPad Software Inc., CA, USA). The difference between two or multiple groups was examined by Student’s *t* test or ANOVA, respectively. A value of *p* < 0.05 was considered to indicate statistical significance.

## Results

### Downregulated SIRT1/FOXO1 pathway in OA cartilage based on clinical specimens

**Fig. 1:**
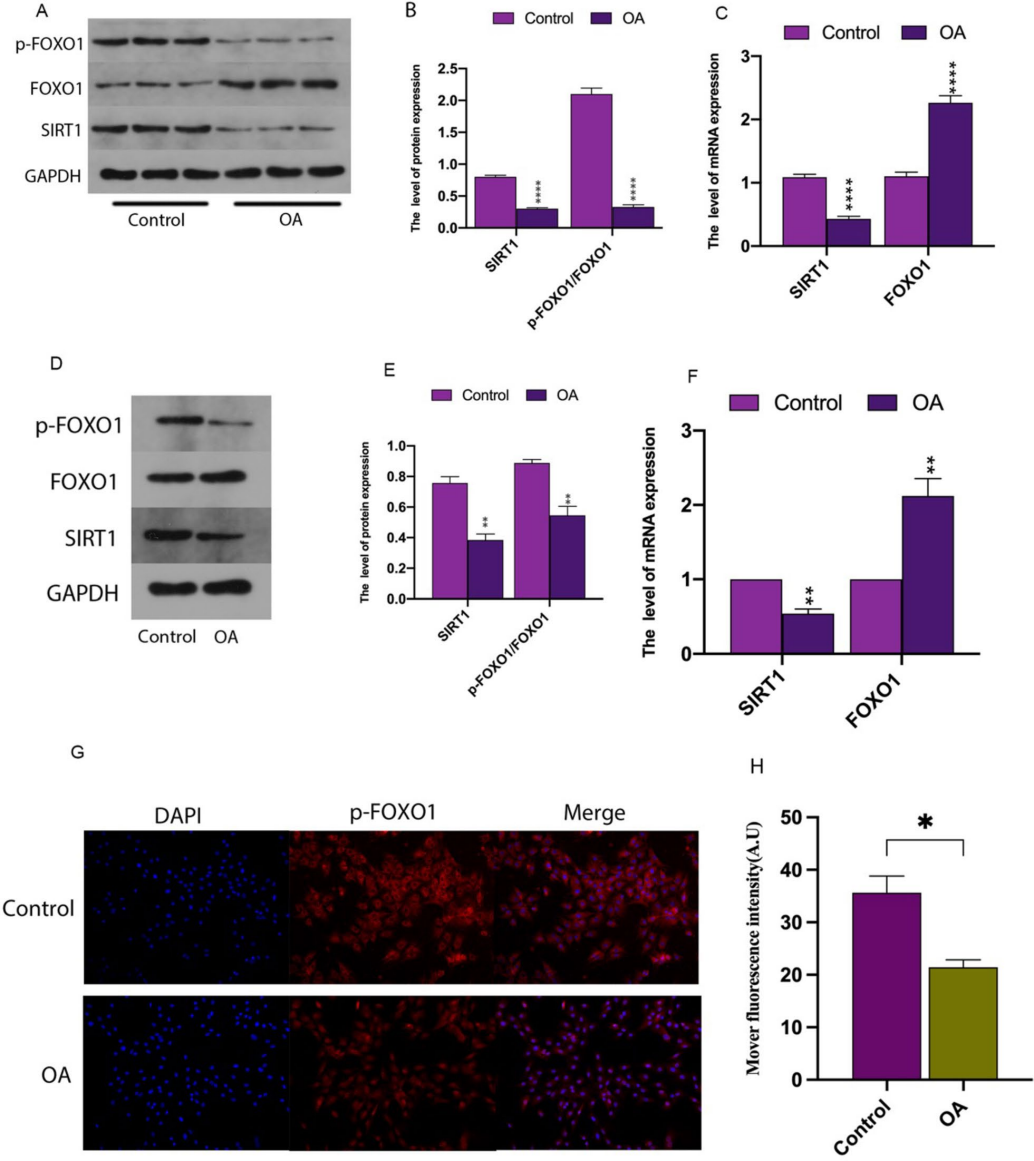
The SIRT1/FOXO1 pathway was downregulated in OA cartilage cells from clinical specimens. **A**, **D** Western blot analysis shows the expression of target proteins SIRT1, FOXO1, and p-FOXO1. **B**, **E** The relative expression of SIRT1, FOXO1, and p-FOXO1 was quantified by normalization with GAPDH. **C**, **F** The mRNA expression levels of SIRT1 and FOXO1 in chondrocytes were quantified by quantitative real-time PCR. **G**, **H** The concentration of P-Foxo1 protein in cartilage tissues was detected by immunofluorescence, and quantitative immunofluorescence analysis was performed using ImageJ software. All data are presented as the mean ± SEM (*n* = 3), *p < 0.05; **p < 0.01; ***p < 0.001; ****p < 0.0001

We examined the expression levels of SIRT1 (Fig. 1A–C), FOXO1 (Fig. 1A–C), and p-FOXO1 (Fig. 1A, B) in normal (*n* = 3) and OA cartilage (*n* = 3) tissues using quantitative real-time PCR, protein blotting, and immunofluorescence techniques to confirm that low activation of the SIRT1/FOXO1 pathway was associated with OA development. Furthermore, chondrocytes were exposed to IL-1β (10 ng/mL) for 24 h to mimic the in vitro OA microenvironment. As shown, the expression levels of SIRT1 (Fig. 1D–F), FOXO1 (Fig. 1D–F), and p-FOXO1 (Fig. 1D, E, G, H) were consistent with the results obtained for OA cartilage. The above results suggest that low expression of the SIRT1/FOXO1 pathway is associated with OA development.

### Effect of RES on chondrocyte proliferation

**Fig. 2:**
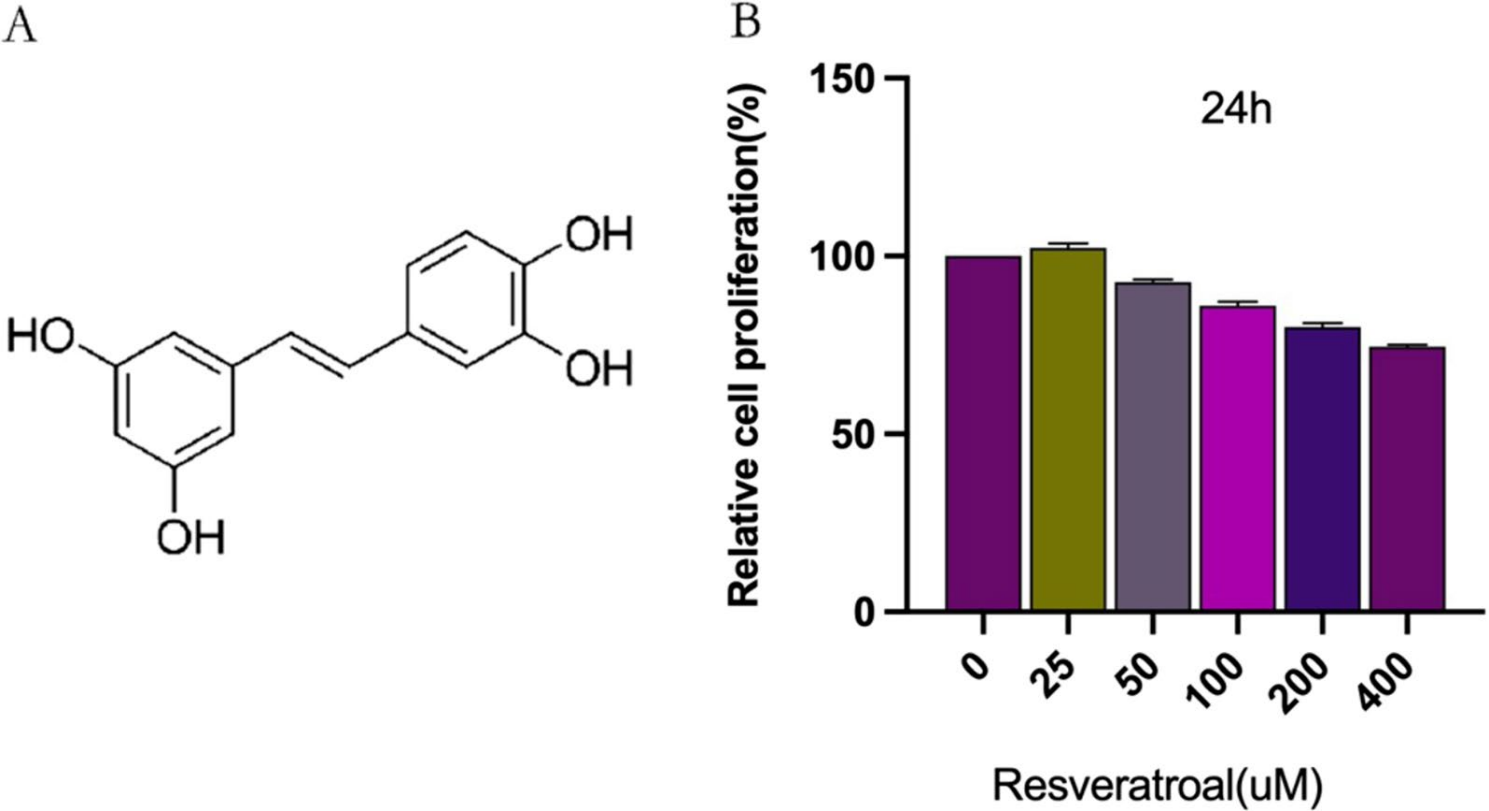
Effect of RES on chondrocyte proliferation. **A** Chemical structure of RES. **B** The effect of RES on chondrocyte proliferation was assayed using CCK-8 to determine the optimal drug concentration for intervening cells, and the RES concentration was selected at 50 µM for all subsequent experiments

The molecular structure of RES is shown in Fig. 2A. The cytotoxicity of RES on chondrocytes was studied using the CCK-8 assay. As demonstrated in Fig. 1B, chondrocytes were treated with different concentrations (0, 25, 50, 100, 200, and 400 µM) of RES for 24 h. The maximum RES concentration not toxic to the cells, defined as cell survival > 90%, was selected. This was found to be 50 µM (Fig. 2B).

### RES attenuates IL-1β-induced chondrocyte damage via the SIRT1/FOXO1 pathway

**Fig. 3:**
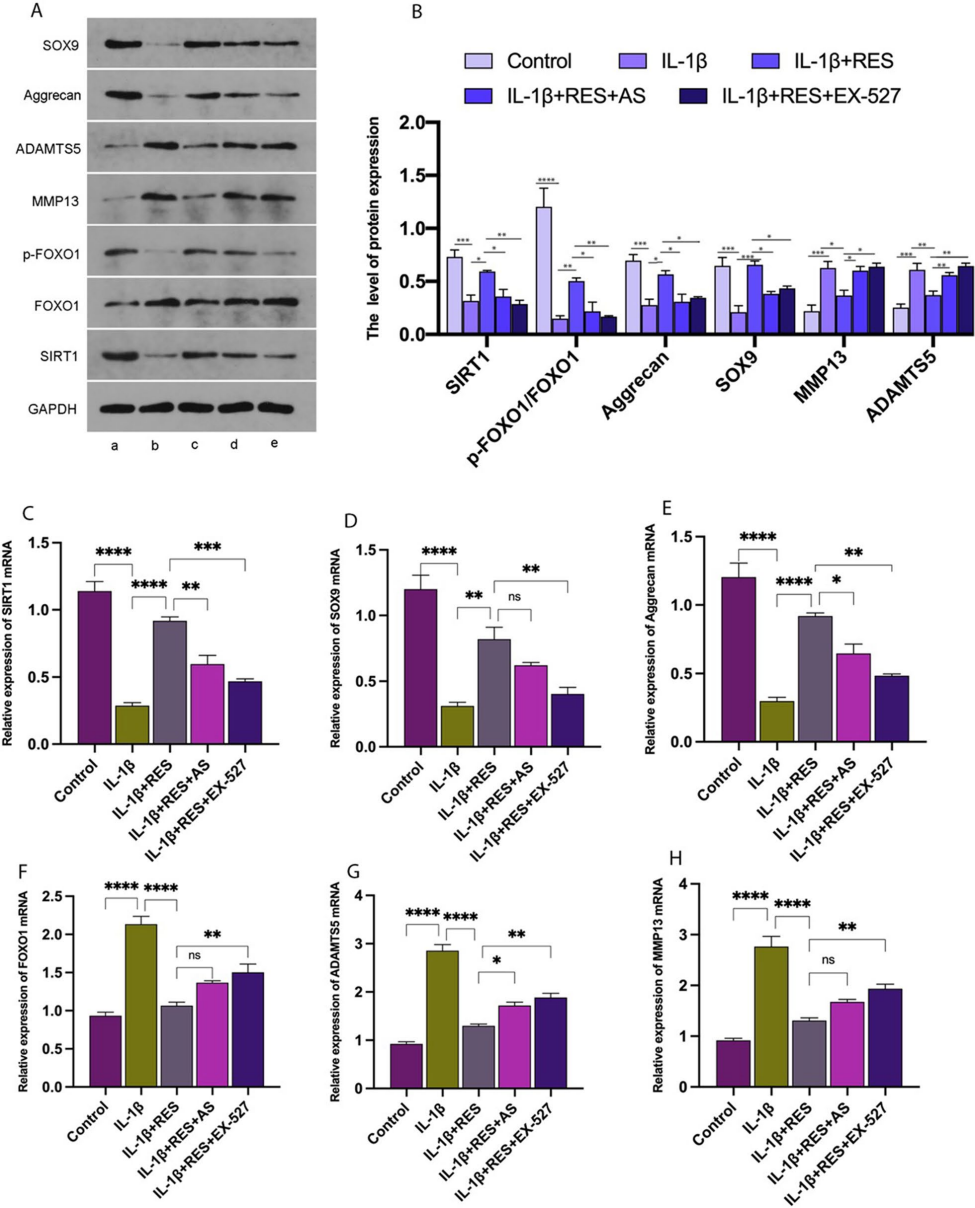
RES attenuates IL-1β-induced chondrocyte damage via the SIRT1/FOXO1 pathway. **A** Western blot shows the expression of target proteins SIRT1, FOXO1, p-FOXO1, aggrecan, SOX9, MMP13, and ADAMTS5, which are associated with the SIRT1/FOXO1 pathway and apoptosis. **B** The relative expression of SIRT1, FOXO1, p-FOXO1, aggrecan, SOX9, MMP13, and ADAMTS5 was quantified by normalization with GAPDH. **C**–**H** The mRNA expression levels of SIRT1, FOXO1, p-FOXO1, aggrecan, SOX9, MMP13, ADAMTS5 in chondrocytes were quantified by quantitative real-time PCR. **a**–**e** Corresponds to control, IL-1β, IL-1β + RES, IL-1β + RES + AS, and IL-1β + RES + EX-527, respectively. AS = FOXO1 inhibitor, EX-527 = SIRT1 inhibitor. All data are presented as the mean ± SEM (*n* = 3), *p < 0.05; **p < 0.01; ***p < 0.001; ****p < 0.0001

Our study found that RES activated SIRT1 in IL-1β cultured chondrocytes and increased p-FOXO1 and FOXO1 expression. When IL-1β-cultured cells were subjected to RES, AS, or RES and EX-527, SIRT1 and p-FOXO1 expression levels were inhibited at the protein and mRNA levels. Surprisingly, after the addition of EX-527, SIRT1 and p-FOXO1protein expression levels were inhibited to a certain extent in the chondrocytes, suggesting an inter-regulatory relationship between SIRT1 and FOXO1. We further investigated the effect of RES on IL-1β-induced chondrocytes by examining the expression of MMP13, ADAMTS5, aggrecan, and SOX9. Quantitative real-time PCR and western blot results showed that protein and mRNA levels of aggrecan (Fig. 3A, B, E) and SOX9 (Fig. 3A, B, D) were significantly decreased in IL-1β-induced chondrocytes compared to controls; mRNA and protein expression levels of MMP13 (Fig. 3A, B, H) and ADAMTS5 (Fig. 3A, B, G) were increased. Compared to IL-1β-induced chondrocytes, the RES group (RES + IL-1β) showed a marked enhancement in the protein and mRNA expression levels of aggrecan (Fig. 3A, B, E) and SOX9 (Fig. 3A, B, D). RES markedly decreased the protein and mRNA expression levels of MMP13 (Fig. 3A, B, H) and ADAMTS5 (Fig. 3A, B, G), attributed to the successful attenuation of IL-1β-induced ECM breakdown in chondrocytes. Compared to the RES group, the addition of the SIRT1 inhibitor EX-527 or the FOXO1 inhibitor AS to the RES group significantly reduced protein and mRNA levels of aggrecan (Fig. 3A, B, E) and SOX9 (Fig. 3A, B, D) and increased the mRNA and protein levels of MMP13 (Fig. 3A, B, H) and ADAMTS5 (Fig. 3A, B, G). The protective effect of RES on chondrocytes was significantly diminished. Thus, RES effectively alleviated IL-1β-induced chondrocyte damage and maintained chondrocyte homeostasis via the SIRT1/FOXO1 pathway.

### Resveratrol improved IL-1β-induced autophagy in chondrocytes via the SIRT1/FOXO1 pathway

**Fig. 4:**
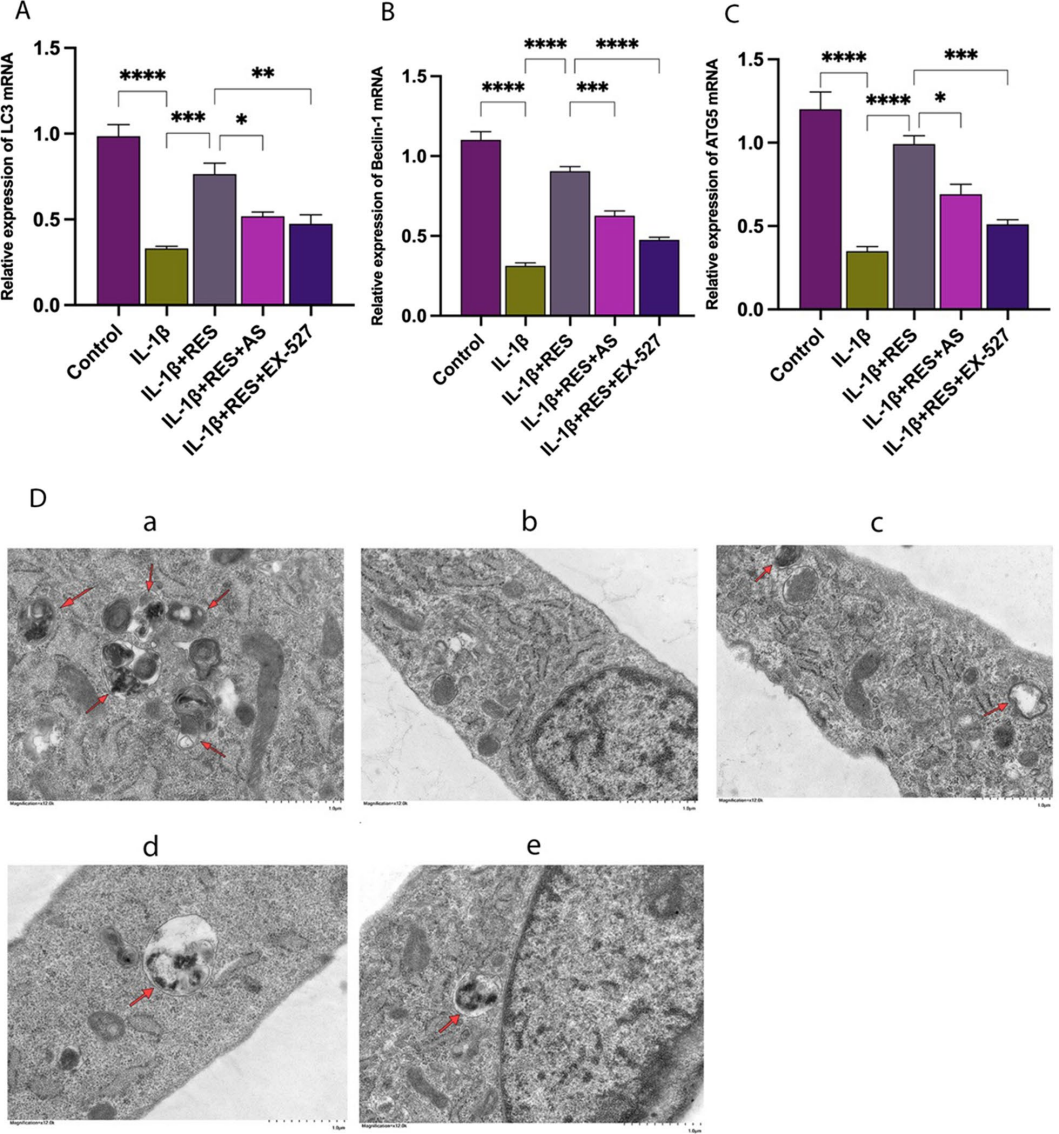
RES improves IL-1β-induced autophagy in chondrocytes via the SIRT1/FOXO1 pathway. **A**–**C** Expression of LC3, ATG5, and Beclin 1 genes associated with chondrocyte autophagy was quantified using quantitative real-time PCR. **D** Groups of intracellular autophagic vesicles were observed by transmission electron microscopy. **a**–**e** Correspond to control, IL-1β, IL-1β + RES, IL-1β + RES + AS, and IL-1β + RES + EX-527, respectively. All data are presented as the mean ± SEM (*n* = 3), *p < 0.05; **p < 0.01; ***p < 0.001; ****p < 0.0001

We used real-time PCR and TEM to investigate the impact of RES on autophagy levels in IL-1β-group chondrocytes. The mRNA levels of autophagy-related genes LC3 (Fig. 4A), ATG5 (Fig. 4C), and Beclin 1 (Fig. 4B) were markedly decreased in IL-1β-induced chondrocytes compared to normal chondrocytes. The RES group (RES + IL-1β) demonstrated significantly increased expression levels of autophagy-related genes in IL-1β-induced chondrocytes compared to the IL-1β group. The addition of EX-527 or AS to the RES group significantly reduced the upregulation of IL-1β-induced autophagy-related genes in chondrocytes by RES compared to the RES group. TEM imaging showed that cells in the IL-1β group had fewer autophagosomes than the control group. Cells in the RES group showed more autophagosomes than the IL-1β-treated group, and when the SIRT1 inhibitor or FOXO1 inhibitor was added, the number of autophagosomes was significantly reduced (Fig. 4D). The above results suggest that RES promotes the restoration of chondrocyte autophagy levels through the SIRT1/FOXO1 pathway.

### Resveratrol attenuates IL-1β-induced apoptosis in chondrocytes via the SIRT1/FOXO1 pathway

**Fig. 5:**
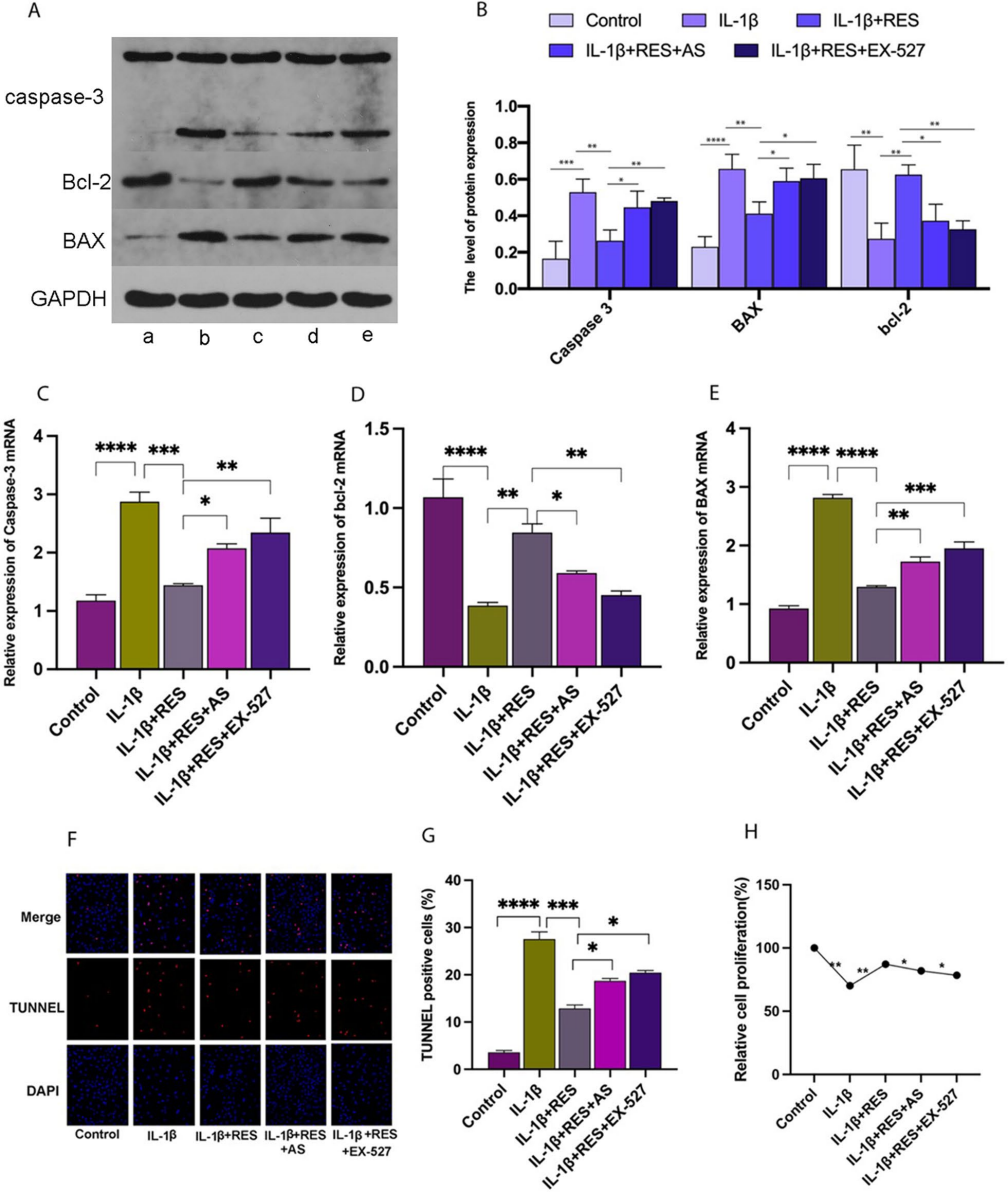
Effect of RES on IL-1β-induced apoptosis levels in chondrocytes. **A** Western blot analysis shows the expression of BCL-2, BAX, caspase-3, genes associated with chondrocyte apoptosis. **B** The relative expression of BCL-2, BAX, and caspase-3 was quantified via normalization to GAPDH. **a**–**e** Correspond to control, IL-1β, IL-1β + RES, IL-1β + RES + AS, and IL-1β + RES + EX-527, respectively. **C**–**E** Levels of caspase-3, BAX, and BCL-2 in chondrocytes were quantified using quantitative real-time PCR. **H** Detection of chondrocyte proliferation in each group using CCK-8. **F**, **G** Detection of chondrocyte proliferation in each group using TUNEL. All data are presented as the mean ± SEM (*n* = 3), *p < 0.05; **p < 0.01; ***p < 0.001; ****p < 0.0001

To analyze the effect of RES on chondrocyte apoptosis, we used real-time PCR, western blot analysis, and TUNEL analysis. The quantitative real-time PCR and western blot results showed that the mRNA and protein levels of apoptosis-related genes caspase-3 (Fig. 5A–C) and BCL-2 (Fig. 5 A, B, D) were significantly increased in the IL-1β group compared to normal chondrocytes. In contrast, the expression of the apoptosis-inhibiting gene BAX (Fig. 5 A, B, E) was decreased. Compared to the IL-1β group, the RES group (RES + IL-1β) showed a significantly attenuated increase in IL-1β-induced apoptosis in chondrocytes. The addition of EX-527 or AS to the RES group significantly increased the level of IL-1β-induced chondrocyte apoptosis compared to the RES group. We performed TUNEL (Fig. 5F, G) experiments to study chondrocyte apoptosis. The CCK-8 results (Fig. 5H) also showed that RES could promote chondrocyte proliferation, consistent with the above findings. The results suggest that RES can inhibit IL-1β-induced chondrocyte apoptosis via the SIRT1/FOXO1 pathway.

## Discussion

We collected tissue samples from three patients with OA and three with normal clinical cartilage tissues. The analysis verified that the SIRT1/FOXO1 pathway was downregulated in OA articular cartilage. RES reversed the imbalance between IL-1β-induced disruption of cartilage ECM metabolism and chondrocyte autophagy and apoptosis by activating the SIRT1/FOXO1 signaling pathway. The data supported the hypothesis that RES can benefit OA chondrocytes by activating the SIRT1/FOXO1 pathway.

According to extensive research, OA cannot be solely attributed to mechanical wear and tear but is also a chronic inflammatory disease [15]. The levels of various pro-inflammatory factors like IL-1β, IL-8, TNF-α, and IL-6 increase in OA joint tissues, with IL-1β being the most critical pro-inflammatory factor [16, 17]. IL-1β levels continue to rise in joint tissues as OA progresses [18]. In this study, IL-1β was used as an inducer of chondrocyte degeneration to simulate the in vivo cartilage degeneration model in patients with OA, like in other studies [19, 20].

The SIRT1/FOXO1 pathway plays an important role in cell metabolism, but there are few studies on the effect of the SIRT1/FOXO1 pathway on OA chondrocytes. Previous studies have demonstrated the beneficial effects of SIRT1 activation on the energy metabolism of OA chondrocytes [21]. Activation of SIRT1 by RES ameliorates palmitate-induced inflammation and reduces OA inflammatory damage [22, 23]. It has been demonstrated that in skeletal muscle and adipocytes, SIRT1 regulates the expression of FOXO1 [24–27]. FOXO1 is highly expressed in chondrocytes and belongs to the FOXO family of transcription factors [28]. Similarly, this study provides convincing evidence that the SIRT1/FOXO1 pathway plays a key role in chondrocytes and may mediate OA development. SIRT1 and P-FOXO1 expression levels were significantly reduced in the articular cartilage cells from OA patients cultured with IL-1β. In addition, ECM degradation was increased considerably in IL-1β-treated chondrocytes compared with normal chondrocytes. After adding RES, the expression levels of SIRT1 and p-FOXO1 increased significantly, and the expression levels of ECM degradation-related genes decreased significantly. In addition, we found that EX-537 could inhibit SIRT1 and downregulate the expression of SIRT1 and p-FOXO1 protein. In contrast, SIRT1 protein expression was not changed after AS blocked FOXO1, but the expression level of p-FOXO1 was affected to some extent. The results further supported that SIRT1 can modulate FOXO1 activity and ameliorate IL-1β-induced chondrocyte damage.

Autophagy promotes the renewal of organelles by engulfing damaged proteins and organelles to achieve efficient cell metabolism [29]. When cells are stressed by hypoxia, starvation, or inflammation, cellular autophagy is triggered to avoid apoptosis and maintain the cell’s metabolic homeostasis [30]. Sasaki et al. showed that autophagy was significantly inhibited in chondrocytes of OA cartilage [31]. The SIRT1/FOXO1 pathway is important for regulating autophagy in vascular endothelial cells [32]. SIRT1/FOXO1 pathway-induced autophagy has been reported to facilitate the survival of energy-deficient cardiomyocytes [33]. We, therefore, explored the link between the SIRT1/FOXO1 pathway and chondrocyte autophagy and apoptosis. We demonstrated that the expression of autophagy-related genes was significantly reduced, and apoptosis-related gene expression was increased in IL-1β-induced chondrocytes. This resulted in a significant imbalance in chondrocyte autophagy and apoptosis levels, which was reversed when RES was added. Furthermore, the inhibited SIRT1 or suppressed FOXO1 expression significantly attenuated the RES reversal of IL-1β-induced imbalance in chondrocyte autophagy and apoptosis levels. This implied that RES might regulate the balance of apoptosis and autophagy levels in OA chondrocytes via the SIRT1/FOXO1 pathway, thereby delaying the progression of OA.

## Conclusion

The results of our clinical specimen experiments confirm the relevance of the SIRT1/FOXO1 pathway in OA pathology. We demonstrated that RES could exert beneficial effects on OA chondrocytes by activating the SIRT1/FOXO1 pathway, which has the following effects: (1) mitigating ECM breakdown in OA chondrocytes; (2) promoting the restoration of autophagy in OA chondrocytes; and (3) inhibiting OA chondrocyte apoptosis. The above results suggest that RES may be a meaningful drug for the prevention and treatment of OA.

## Data Availability

The raw data supporting the conclusions of this manuscript will be made available by the authors, without undue reservation, to any qualified researcher.

## References

[1] Hunter DJ, Bierma-Zeinstra S (2019). Osteoarthritis. Lancet.

[2] Ishidou Y, Matsuyama K, Sakuma D, Setoguchi T, Nagano S, Kawamura I, Maeda S, Komiya S (2017). Osteoarthritis of the hip joint in elderly patients is most commonly atrophic, with low parameters of acetabular dysplasia and possible involvement of osteoporosis. Arch Osteoporos.

[3] Kang R, Tang D (2012). Autophagy in pancreatic cancer pathogenesis and treatment. Am J Cancer Res.

[4] Kang S, Kim JE, Song NR, Jung SK, Lee MH, Park JS, Yeom MH, Bode AM, Dong Z, Lee KW (2014). The ginsenoside 20-O-beta-D-glucopyranosyl-20(S)-protopanaxadiol induces autophagy and apoptosis in human melanoma via AMPK/JNK phosphorylation. PLoS ONE.

[5] Kong D, Yan Y, He XY, Yang H, Liang B, Wang J, He Y, Ding Y, Yu H (2019). Effects of resveratrol on the mechanisms of antioxidants and estrogen in Alzheimer's disease. Biomed Res Int.

[6] Glasson SS, Askew R, Sheppard B, Carito B, Blanchet T, Ma HL, Flannery CR, Peluso D, Kanki K, Yang Z (2005). Deletion of active ADAMTS5 prevents cartilage degradation in a murine model of osteoarthritis. Nature.

[7] Sun H, Wu Y, Pan Z, Yu D, Chen P, Zhang X, Wu H, Zhang X, An C, Chen Y (2018). Gefitinib for epidermal growth factor receptor activated osteoarthritis subpopulation treatment. EBioMedicine.

[8] Lu H, Zeng C, Chen M, Lian L, Dai Y, Zhao H (2015). Lentiviral vector-mediated over-expression of Sox9 protected chondrocytes from IL-1beta induced degeneration and apoptosis. Int J Clin Exp Pathol.

[9] McNulty MA, Loeser RF, Davey C, Callahan MF, Ferguson CM, Carlson CS (2012). Histopathology of naturally occurring and surgically induced osteoarthritis in mice. Osteoarthr Cartil.

[10] Musumeci G, Castrogiovanni P, Trovato FM, Weinberg AM, Al-Wasiyah MK, Alqahtani MH, Mobasheri A (2015). Biomarkers of chondrocyte apoptosis and autophagy in osteoarthritis. Int J Mol Sci.

[11] Maiuri MC, Zalckvar E, Kimchi A, Kroemer G (2007). Self-eating and self-killing: crosstalk between autophagy and apoptosis. Nat Rev Mol Cell Biol.

[12] Deng Z, Li Y, Liu H, Xiao S, Li L, Tian J, Cheng C, Zhang G, Zhang F (2019). Biosci Rep.

[13] Calnan DR, Brunet A (2008). The FoxO code. Oncogene.

[14] Yang X, Jiang T, Wang Y, Guo L (2019). The role and mechanism of SIRT1 in resveratrol-regulated osteoblast autophagy in osteoporosis rats. Sci Rep.

[15] Robinson WH, Lepus CM, Wang Q, Raghu H, Mao R, Lindstrom TM, Sokolove J (2016). Low-grade inflammation as a key mediator of the pathogenesis of osteoarthritis. Nat Rev Rheumatol.

[16] Kapoor M, Martel-Pelletier J, Lajeunesse D, Pelletier JP, Fahmi H (2011). Role of proinflammatory cytokines in the pathophysiology of osteoarthritis. Nat Rev Rheumatol.

[17] Wojdasiewicz P, Poniatowski LA, Szukiewicz D (2014). The role of inflammatory and anti-inflammatory cytokines in the pathogenesis of osteoarthritis. Mediators Inflamm.

[18] Sun HY, Hu KZ, Yin ZS (2017). Inhibition of the p38-MAPK signaling pathway suppresses the apoptosis and expression of proinflammatory cytokines in human osteoarthritis chondrocytes. Cytokine.

[19] Corciulo C, Lendhey M, Wilder T, Schoen H, Cornelissen AS, Chang G, Kennedy OD, Cronstein BN (2017). Endogenous adenosine maintains cartilage homeostasis and exogenous adenosine inhibits osteoarthritis progression. Nat Commun.

[20] Li K, Zhang Y, Zhang Y, Jiang W, Shen J, Xu S, Cai D, Shen J, Huang B, Li M (2018). Tyrosine kinase Fyn promotes osteoarthritis by activating the beta-catenin pathway. Ann Rheum Dis.

[21] Lu Y, Zhou L, Wang L, He S, Ren H, Zhou N, Hu Z (2020). The role of SIRT1 in BMP2-induced chondrogenic differentiation and cartilage maintenance under oxidative stress. Aging (Albany NY).

[22] Batshon G, Elayyan J, Qiq O, Reich E, Ben-Aderet L, Kandel L, Haze A, Steinmeyer J, Lefebvre V, Zhang H (2020). Serum NT/CT SIRT1 ratio reflects early osteoarthritis and chondrosenescence. Ann Rheum Dis.

[23] Wang C, Yao Z, Zhang Y, Yang Y, Liu J, Shi Y, Zhang C (2020). Metformin mitigates cartilage degradation by activating AMPK/SIRT1-Mediated autophagy in a mouse osteoarthritis model. Front Pharmacol.

[24] Liu X, Zheng H (2021). Modulation of Sirt1 and FoxO1 on hypothalamic leptin-mediated sympathetic activation and inflammation in diet-induced obese rats. J Am Heart Assoc.

[25] Al-Massadi O, Quinones M, Clasadonte J, Hernandez-Bautista R, Romero-Pico A, Folgueira C, Morgan DA, Kallo I, Heras V, Senra A (2019). MCH regulates SIRT1/FoxO1 and reduces POMC neuronal activity to induce hyperphagia, adiposity, and glucose intolerance. Diabetes.

[26] Huang X, Sun J, Chen G, Niu C, Wang Y, Zhao C, Sun J, Huang H, Huang S, Liang Y (2019). Resveratrol promotes diabetic wound healing via SIRT1-FOXO1-c-Myc signaling pathway-mediated angiogenesis. Front Pharmacol.

[27] Wang Y, Zhang L, Che X, Li W, Liu Z, Jiang J (2018). Roles of SIRT1/FoxO1/SREBP-1 in the development of progestin resistance in endometrial cancer. Arch Gynecol Obstet.

[28] Feige JN, Auwerx J (2008). Transcriptional targets of sirtuins in the coordination of mammalian physiology. Curr Opin Cell Biol.

[29] Yin H, Wu H, Chen Y, Zhang J, Zheng M, Chen G, Li L, Lu Q (2018). The therapeutic and pathogenic role of autophagy in autoimmune diseases. Front Immunol.

[30] Saito S, Nakashima A (2013). Review: The role of autophagy in extravillous trophoblast function under hypoxia. Placenta.

[31] Sasaki H, Takayama K, Matsushita T, Ishida K, Kubo S, Matsumoto T, Fujita N, Oka S, Kurosaka M, Kuroda R (2012). Autophagy modulates osteoarthritis-related gene expression in human chondrocytes. Arthritis Rheum.

[32] Lin X, Ouyang S, Zhi C, Li P, Tan X, Ma W, Yu J, Peng T, Chen X, Li L (2021). Focus on ferroptosis, pyroptosis, apoptosis and autophagy of vascular endothelial cells to the strategic targets for the treatment of atherosclerosis. Arch Biochem Biophys.

[33] Hariharan N, Maejima Y, Nakae J, Paik J, Depinho RA, Sadoshima J (2010). Deacetylation of FoxO by Sirt1 plays an essential role in mediating starvation-induced autophagy in cardiac myocytes. Circ Res.

